# Detection of COVID-19 using deep learning on x-ray lung images

**DOI:** 10.7717/peerj-cs.1082

**Published:** 2022-09-07

**Authors:** AbdAlRahman Odeh, Ayah Alomar, Shadi Aljawarneh

**Affiliations:** Faculty of Computer and Information Technology, Jordan University of Science and Technology, Irbid, Jordan

**Keywords:** Deep learning, Supervised learning, Classification, Transfer learning, COVID-19

## Abstract

COVID-19 is a widespread deadly virus that directly affects the human lungs. The spread of COVID-19 did not stop at humans but also reached animals, so it was necessary to limit it is spread and diagnose cases quickly by applying a quarantine to the infected people. Recently x-ray lung images are used to determine the infection and from here the idea of this research came to use deep learning techniques to analyze x-ray lung images publicly available on Kaggle to possibly detect COVID-19 infection. In this article, we have proposed a method to possibly detect the COVID-19 by analyzing the X-ray images and applying a number of deep learning pre-trained models such as InceptionV3, DenseNet121, ResNet50, and VGG16, and the results are compared to determine the best performance model and accuracy with the least loss for our dataset. Our evaluation results showed that the best performing model for our dataset is ResNet50 with accuracies of 99.99%, 99.50%, and 99.44% for training, validation, and testing respectively followed by DenseNet121, InceptionV3, and finally VGG16.

## Introduction

**Figure 1:**
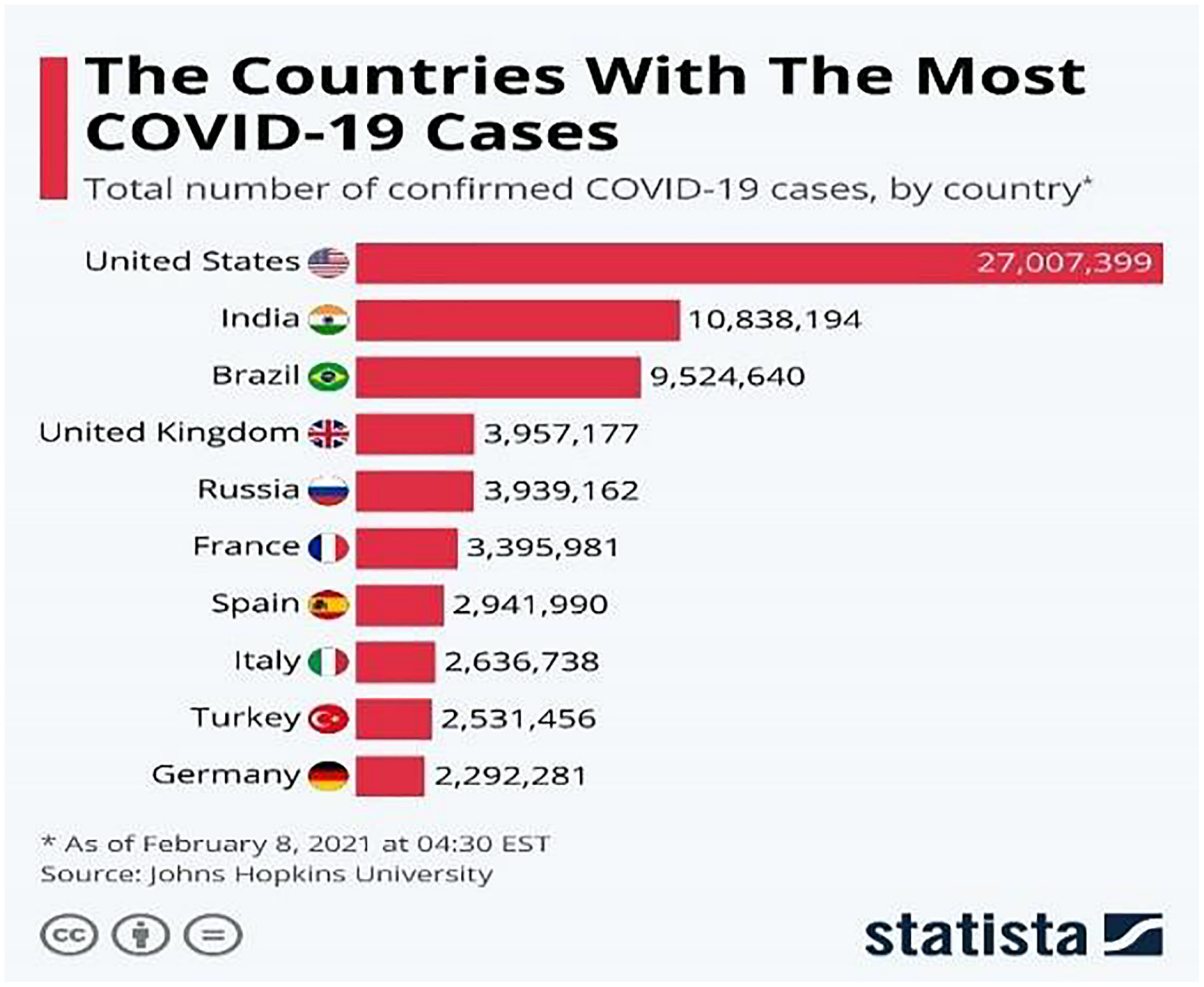
Countries with the most COVID-19 cases as of February 8, 2021.

The COVID-19 (WHO, 2019) virus is a fast-spreading disease that has appeared all over the world; Fig. 1 (Armstrong & Richter, 2021) represents the high spreading of COVID-19 in many countries as of February 8, 2021. It affects all aspects of life, economically, educationally, healthily, socially, and, most dangerously, human health, as it has caused the death of millions of people (Maatuk et al., 2022). There are two ways to control the spread of the virus: one is to take precautions, and the other is to take the vaccinations that are being specifically developed to defend against the disease. Studies have shown that vaccinations could cause allergic responses (Schumaker et al., 2020), thus some people may not be allowed to take it and would lead to the other way which is the early detection of the disease by chest x-ray to develop a treatment plan which provides a great opportunity for recovery and to prevent its spread.

Deep learning (Schulz & Behnke, 2012) has also proven to be useful in various fields including natural language processing (Vasilakes, Zhou & Zhang, 2020), computer vision (Voulodimos et al., 2018), healthcare (Esteva et al., 2019), and many other areas (Chatterjee, 2022). This research focuses on applying different deep learning approaches in the medical field. Many scholars have performed studies to help improve the medical field with computer-aid techniques to help doctors diagnose diseases and plan an early treatment process for the patients because sometimes tests can take a long time to receive results (Ayan & Ünver, 2019). One of these applications is using a convolutional neural networks (CNN) on medical images to extract features and train on them, for instance, using x-ray images (Chakraborty, Murali & Mitra, 2022) or CT-scans (Yadlapalli et al., 2022) to classify a new x-ray image as normal or diseased. As discussed earlier, diagnosing respiratory system diseases may be very useful for both doctors and patients. For this purpose, a dataset of radiography images of a patient’s chest is acquired to train different deep learning. The result of this training is a model that would be able to classify a new chest x-ray as normal or COVID-19. Some researchers have done studies on the diagnosis of COVID-19 (Padma & Kumari, 2020), lung opacity, and pneumonia infection (Kundu et al., 2021) in the x-rays while others have investigated all these diseases together (Khan et al., 2022).

Different approaches are provided in deep learning such as transfer learning. Many networks will be trained on huge datasets and have preserved the weights and parameters of those datasets. Transfer learning is defined as when using a pre-trained model or network for solving a specific task by freezing the earlier layers to preserve the pre-trained weights and adding some layers in the end for classification purposes and training some weights for the specific task (Bozinovski, 2020).

In this article, a dataset containing a set of lung X-ray images associated with corona disease was studied. Therefore, deep learning was chosen to train different neural networks and models including pre-trained ones on the images to determine whether the person was infected by COVID-19 or not and provide the needed assistance for them. Furthermore, the effects of manipulating and fine-tuning the hyperparameters of the networks are explored in addition to changing the architecture of the network to achieve satisfying and better results.

The rest of this article is organized as follows: the related work section discusses related work on deep learning and detecting diseases. The methodology section describes the proposed methods. The results section represents the results of the classification. The discussion section examines the results and interprets them. Finally, the conclusions and future work section to point out possible areas of research.

## Related work

Deep learning has been used in different fields including the health sector. It is used to detect different kinds of diseases from x-rays, symptoms, or CT-Scans. Some of the research in this field is discussed and to be more specific, the research to detect respiratory diseases and COVID-19 is discussed.

For starters, the authors of Chakraborty, Murali & Mitra (2022), where another approach is presented for COVID-19 detection using deep learning. They stated that their system is for COVID-19 detection, but similar to Aggarwal et al. (2022) they classify the image into three classes. They used a larger dataset than the previous one and which contains 10,040 samples and has some imbalance issues. Still, they attempted to remedy the sample size issue through data augmentation as a data pre-processing step in addition to resizing the images to be fit for the model. The pre-trained models such as AlexNet, VGG, ResNet, and DenseNet were used for the comparison, but they have not mentioned any fine-tuning process for these networks in their work. The main strength of this article is using image segmentation to focus on the required area of the image which enhances the training quality and results of the model. The experiments were conducted using 70% training, 10% validation, and 20% testing along with 200 epochs and 50 batch sizes. The evaluation metrics that were taken into account were confusion matrix, accuracy, F1-score, precision, and recall in addition to the ROC curve. In the results section, the proposed model achieved a detection accuracy of 96.43% and a sensitivity of 93.68%. However, the sizes of the dataset even with data augmentation are not sufficient, it still needs more samples. Furthermore, none of hyperparameters comparison and nor fine-tuning of the networks were depicted.

Another research in this area was conducted by Yadlapalli et al. (2022) who studied the classification of CT scans for the diagnosis of COVID-19 patients. Their approach was to use K-means clustering as an image segmentation technique to separate the area of interest from the background and then feed the segmented images to the VGG16 network and another three-layer CNN built from scratch. The image segmentation led to smoother learning for both models even though it led to some kind of overfitting as it reduces the number of features in an image. A COVID-19-CT dataset was used that is distributed as 349 positive and 397 negative scans. For evaluation, they took into consideration the accuracy, F1-score, and area under the curve (AUC) for each model and their results showed that VGG16 achieved the highest results with an accuracy of 89%, F1-Score of 88%, and AUC OF 0.94 when compared with other networks.

Padma & Kumari (2020) built a CNN from scratch to identify COVID-19 patients. The motivation was to prevent further spread and initiate earlier treatment for existing cases. The dataset was acquired from open sources like GitHub and Kaggle and consists of 60 images where 30 of which are of COVID-19 patients and 30 of normal patients. The network used CNN layers, max-pooling to reduce dimensionalities, dropout to reduce the number of parameters, flattening to convert the image into a one-dimensional vector, and finally, a classification layer where the output is binary either positive or negative. ReLU was used as an activation function for the CNN layer which enhanced the performance by 2.5%. Furthermore, the dataset was split into 80:20 for training and testing. A training accuracy of 99.2% was acquired, with a validation accuracy of 98.8% and a loss of 0.3%. The precision of their study was 100% indicating that their model has no false-positive values.

Khan et al. (2022) compared the performance of three different pre-trained networks and used two strategies, where they added some regularization techniques to enhance the performance of COVID-19 detection. The dataset they used in their research comes from different sources such as Kaggle and GitHub and consists of 21,165 images distributed over four classes that are normal, lung opacity, viral pneumonia, and COVID-19. They mentioned that the dataset was imbalanced and used data augmentation to make it more balanced, but this only works to a certain extent, and collecting more data works better than this approach. They applied rotation, and horizontal and vertical flipping to produce more samples for their dataset in addition to rescaling and resizing the images as part of the pre-processing steps of their work. The split ratio of the dataset is 70:20:10. The comparison includes fine-tuning the hyperparameters of the networks (such as dropout percentage and learning rate) that are not clear. The results showed that EfficientNetB1 outperforms the other models, and that strategy II achieves better performance than strategy I. The recorded accuracy of the best performing model was 96.13% after choosing the confusion matrix, accuracy, F1-score, precision, and recall as the evaluation metrics.

Aggarwal et al. (2022) proposed a comparison between eight fine-tuned neural networks to classify x-ray images to different diseases represented as classes. Their work included working on two datasets where the first one consisted of three classes: normal, COVID-19, and pneumonia with a total of 709 samples, whereas the second dataset contains the same classes but focuses on the pneumonia class and divides it into bacterial and viral and consists of 959 samples. For experimenting, the datasets were split into 80% for training and 20% for testing. Because the sizes of the datasets are small, some data augmentation techniques are implemented to increase the sample size and make the model more generalizable. Furthermore, some preprocessing was done on the data such as applying CLAHE followed by image scaling and resizing. The metrics for evaluation that were used in this article include confusion matrix, accuracy, F1-score, precision, and recall. The acquired results showed that DenseNet121 achieved the best accuracy for the first dataset (97%) and the second dataset MobileNetV2 came (with 81%) with the best accuracy. The weaknesses and limitations of such a article are the sizes of the dataset even with data augmentation, it still needs more samples. In addition, the acquired results for bacterial and viral pneumonia were poor in terms of F1-score when compared with the results of the other classes and need further enchantments because samples of viral and bacterial pneumonia have been misclassified into other categories mainly into the COVID-19 category. Lastly, the fine-tuned architecture of the networks is not discussed and no experimenting with the hyperparameters is shown, which needs to be shown and discussed when doing comparative research.

Some researchers such as Nazish et al. (2021) trained their machine learning algorithms using x-ray images of the lungs for the classification into either COVID-19 or normal lungs. X-rays of normal lung and COIVID-19 patients were captured and connected to a dataset on Kaggle. They used these images and applied some pre-processing to them like removing noise and resizing. After that, they extracted features from the images using histogram oriented gradient (HOG). The dataset was split into 70% for training and the rest of 30% for testing. Two classifiers were applied to the extracted features which were SVM and logistic regression (LR). Their results have shown that the SVM has performed with better accuracy than LR with accuracies of 96% and 92% respectively.

From the literature that is discussed, the size of the dataset is the main issue that faces most researchers. In addition, most of these articles do not demonstrate a comparison of hyperparameters of the networks or how the pre-trained networks are fine-tuned for the specific intended task. Furthermore, a lot of the literature discusses respiratory diseases in general but a specialized model for detecting COVID-19 cases from x-ray images is needed instead of a model that can predict different diseases with less efficiency and accuracy. Some techniques that were used such as data augmentation, segmentation, and masking might come in handy and be used in our research to get better performance and results from the model.

## Methods

### Overview

**Figure 2:**
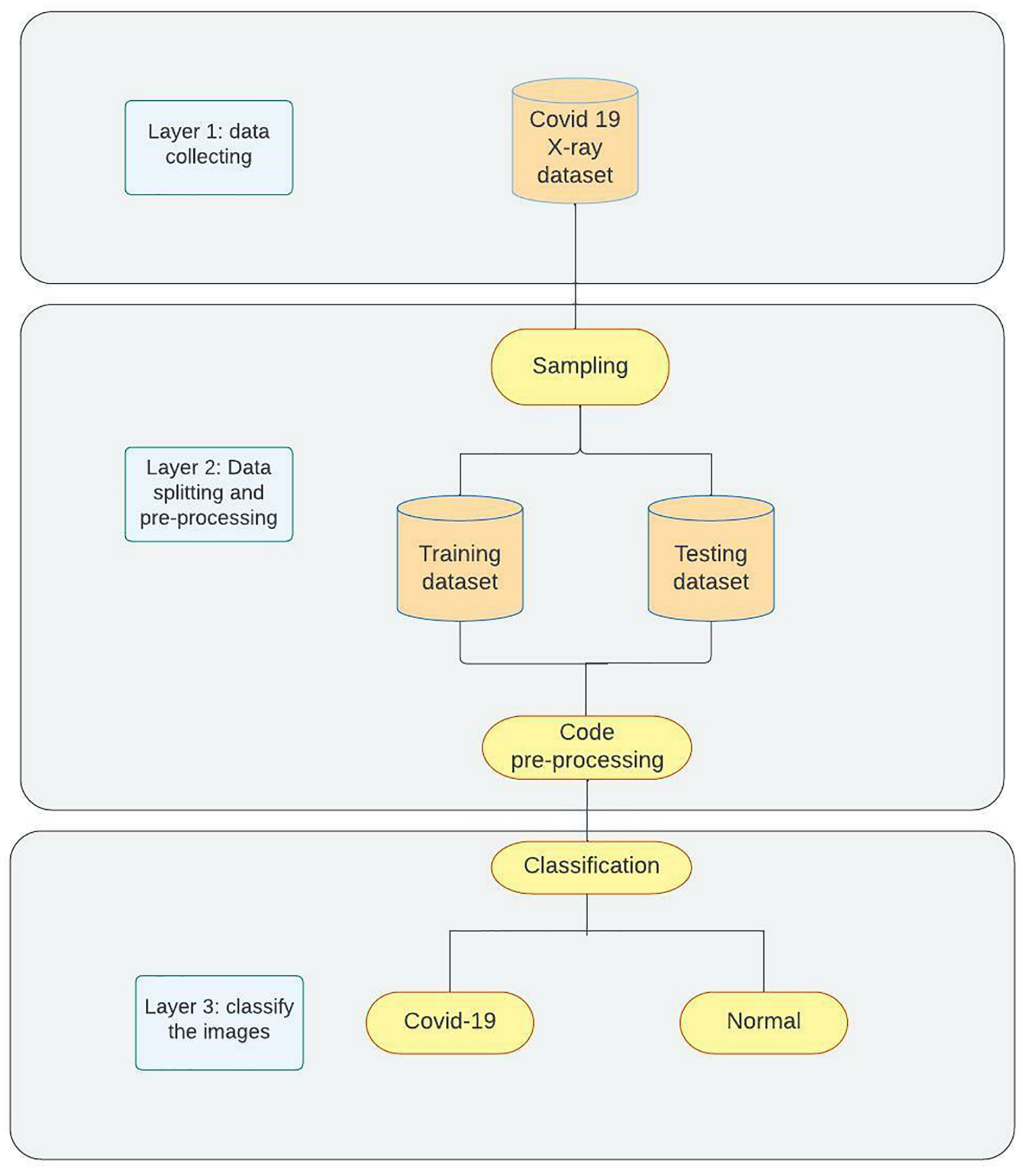
Methodology overview.

The proposed model for detecting COVID-19 as shown in Fig. 2 contains three phases. Phase 1 demonstrates the data collection process and the manual pre-processing that is used to change image formats and sizes. Phase 2 represents sampling and splitting of the dataset into training and testing datasets followed by applying code pre-processing on the data to prepare them for Phase 3 where the models are trained on the training data then tested on the testing data and classification networks will be applied to classify the images into two categories (COVID-19, Normal). For further clarification of our approach, it is as follows: Data Collection (Kaggle); Data Preparation (resizing and renaming); Data Splitting; Data Preprocessing (depending on the network); Training; Evaluating (accuracy, loss, and confusion matrix).

### Dataset

Two publicly available datasets on Kaggle were used and merged in this research. The COVID-19 Radiography Dataset (Rahman, 2022) consists of 21,178 images of lung x-rays distributed over four classes that are normal, viral pneumonia, lung opacity, and COVID-19. The second dataset was called COVID-19 X-ray (Vantaggiato, 2021), which has 2,133 categorized into three categories which are normal, pneumonia, and COVID-19. Because only COVID-19 disease is discussed in our work, the images of the other classes are discarded. Table 1 shows the distribution of the data over all the classes of the two datasets and Table 2 shows the distributions after discarding irrelevant classes.

**Table 1:** Original dataset distribution.

Class	Dataset 1	Dataset 2
Normal	10,192	711
Lung opacity	6,012	–
Pneumonia	1,345	711
COVID	3,616	711
Total	21,178	2,133

**Table 2:** Distribution of data after filtering.

Class	Dataset 1	Dataset 2	Full dataset
Normal	10,192	711	10,903
COVID	3,616	711	4,327
Total	13,808	1,422	15,230

### Pre-processing and splitting

Several steps are taken into consideration when data preparation for the model is required. In this article, four main steps are applied to the acquired dataset to prepare them for training. The first step was to resize all the acquired images into (224 × 224 × 1) images. The value 224 was chosen for the number of pixels as all the pre-trained networks that were used in this article have been trained on the ImageNet dataset that has the size of 224 × 224 and because the images are x-rayed images, then changing the number of channels in the images to one making them in greyscale is necessary.

After resizing the images, the images were renamed because some did not have meaningful names. For that reason, all the images inside a specific folder were renamed to the category that they belong to. It is known in deep learning that the model trains on a set of data, then it is validated on another set and finally is tested on totally new data, thus data splitting comes in handy to help to achieve this flow of training process of deep learning models. Furthermore, it was noticed from the distribution table in the previous section that the data was imbalanced to some extent. To overcome this issue, some of the previous work discussed in the related work section has used data augmentation techniques to create more samples of the class that has fewer samples. That being said, no data augmentation was applied in our study because after trying several parameters of augmentation that are used in previous research (Ayan & Ünver, 2019; Irfan et al., 2020), all the results of the training and testing got worse. That means the networks performed better without data augmentation.

Nevertheless, the imbalanced data problem still exists and needs to be dealt with. The other approach to solve this challenge is to select a specific number of samples for each class where the number of samples may differ, but they should be close to each other. Several splitting combinations are tried to overcome the imbalance issue in the data set like 1,500, 2,000, 3,000, and 3,500 for the training set and the rest for validation and testing. The best split size that received good results is 3,500 for COVID and 5,000 for the normal group for the training and 200 for validation and 626 for testing and the rest is discarded. So, in this research, the results that are provided are done on these splitting sizes which are done randomly, and Table 3 shows the distribution of the images across the three types of datasets. The final step of pre-processing is done when the code loads the data from the files. Several already defined pre-processing functions are offered by Keras for each model. Each function performs the pre-processing steps on the data that are necessary to prepare the data for that specific model this includes rescaling the pixel values, applying PCA, normalization using mean, or any other pre-processing techniques. By the end of this process, the data are resized, renamed, split, pre-processed, and ready for the next step which is training the model.

**Table 3:** Distribution of data after splitting.

Class	Dataset 1	Dataset 2	Full dataset
Normal	10,192	711	10,903
Class	Training	Validation	Testing
Normal	5,000	200	626
COVID	3,500	200	626
Total	8,500	400	1,252

### Transfer learning

As mentioned in the introduction section, transfer learning (Bozinovski, 2020) refers to using pre-trained networks on a large dataset as the foundation for creating a new network architecture that may be fine-tuned and used on a new dataset for a specific task. This allows the use of weights that were obtained during the training process on the large dataset and requires fewer data to be trained on for the new task, which makes training the network easier and more efficient. Transfer learning models are widely used with pretrained models for large and complex picture classification competitions, such as the ImageNet competition.

In this study, pre-trained ResNet (He et al., 2016), VGG16 (Simonyan & Zisserman, 2015), Inception (Szegedy et al., 2016), and DenseNet (Huang et al., 2017) models were used. These models proved to be the best models to be used in medical image applications (Mallick, 2021). The concept of transfer learning was based on freezing the weights of the layers that were not used to train while unfreezing the other layers.

Each one of the chosen networks introduced a new approach and architecture for the training process. VGG16 offers a shallow architecture with 16 layers of convolutional, drop-out, and max-pooling layers. It was the winner of the ImageNet competition in 2014. As it is considered one of the early networks, no special architecture is implemented. InceptionV3 introduces the inception module to the architecture with 48 blocks of layers and the early versions of it have also won the competition of ImageNet of 2014 alongside VGG16. After that, ResNet dominated the competition in 2015 with noticeable improvement in the results by introducing residual blocks and skip connection concept with 50 layers. The final and one of the most recent networks is DenseNet. It is based on a dense blocks approach that allows for developing deeper networks and improving performance. In this research, the version of 121 layers is chosen as it is the most suitable and recommended one from previous research.

Research has proven that the best way to fine-tune the transfer learning network for lung x-ray images is through freezing the first two blocks of layers of the network and training the rest of the blocks (Raghu et al., 2019). Thus, this method is applied in this article in addition to cutting off the output layer of the pre-trained networks and replacing it with a new classifier layer that uses the Sigmoid activation function to classify the images of the dataset of the specific task. After that, the networks are trained on the pre-processed training dataset and evaluated on the testing dataset. The results of the conducted experiments are presented in the next section.

### Experiments and evaluation

**Figure 3:**
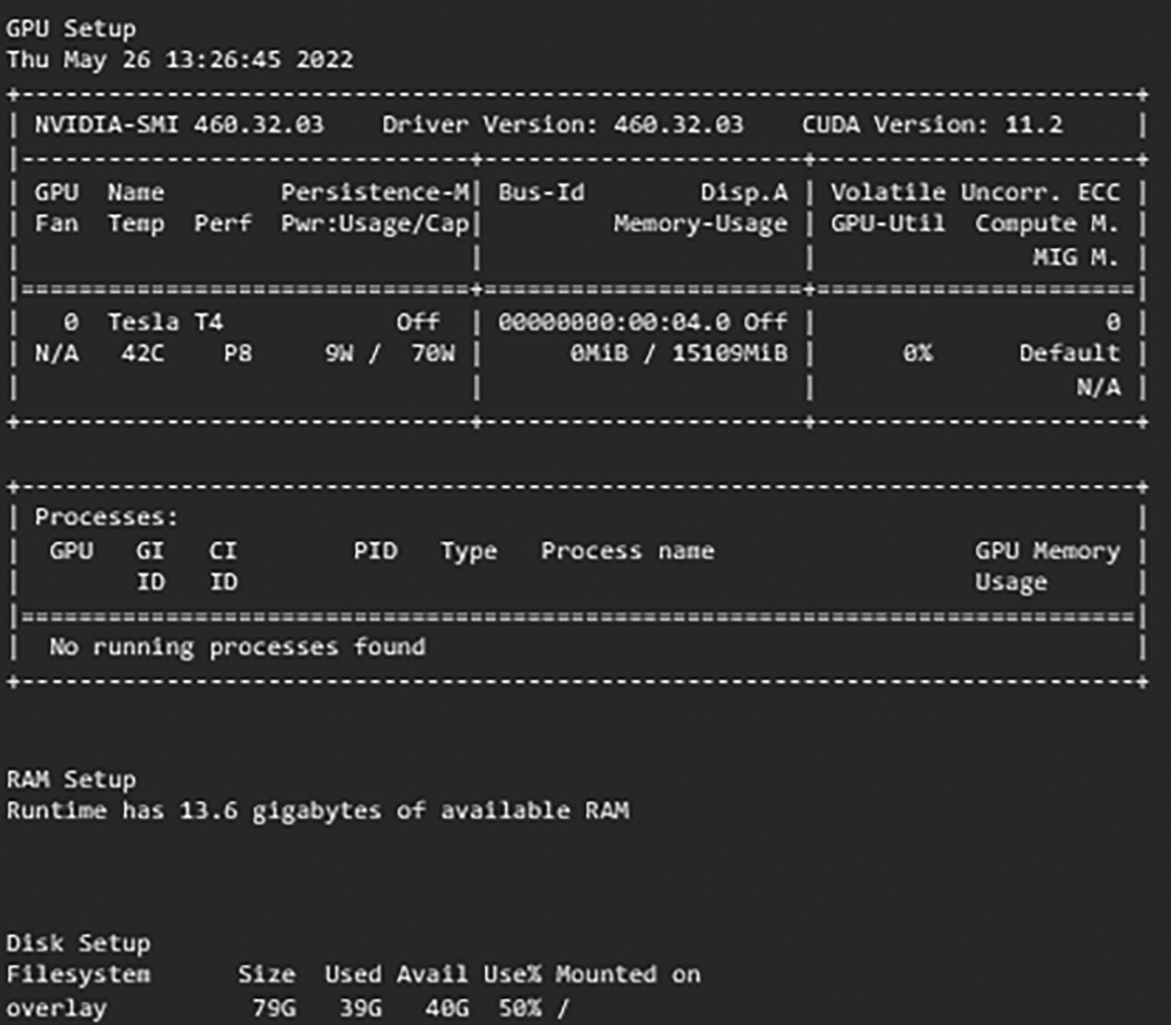
Environment configurations.

After setting and implementing the architecture for the networks, it is time to run experiments on them. Many hyperparameters can affect the learning process of the network like the optimization function, activation function, learning rate, batch size, dropout percentage, and others. In our work, experiments are run with different values for learning rate, dropout percentage, batch sizes, and decay for learning rate. Table 4 shows the settings of the experiment. The first experiment included running each model with both learning rates and then choosing the one with better results. After that, another experiment with the decay values and freezing the learning rate to the one chosen earlier and comparing the results. The experiments for the dropout percentage and batch size are conducted the same way. The results and comparison are provided in the results and discussion sections. Adam optimizer is used as the optimization function for the network and early stopping is implemented with the patience of five along with 50 epochs it monitors the validation loss and returns the weights of the best epoch. For evaluating the developed models, several metrics are defined. The metrics that are used are the weighted accuracy, precision, recall, F1-score, confusion matrix, and learning curves for each model. Furthermore, the free version of Google Colab is used for conducting these experiments. It provides users with 13 GB of RAM. Tesla T4 P8 GPU, and 78 GB of storage with a maximum runtime of 6 hours that is shown in Fig. 3.

**Table 4:** Expirement settings.

Parameter	Value
Learning rate	0.001/0.0001
Decay	Yes/No
Dropout percentage	0.5/0.2
Batch size	128/64/32

## Results

In this section, the results of the experiments are presented and compared to leverage one of the networks to be the best-suited network for our dataset and use case. The comparisons are based on the training, validation, and testing loss. The network with the lowest testing loss and close to the validation and training losses is considered to be the best one as it is proof that the network does not overfit or underfit. Furthermore, in case of two networks have the same losses, comparisons are expanded to include reports such as classification report data and confusion matrix evaluation to determine the best one. This section is divided into two subsections which are the results of each transfer learning network where different comparisons on hyperparameters values are conducted to choose the best set of hyperparameters for each network. The second subsection compares the networks with the best set of hyperparameters of different types of networks to determine the best overall network. A detailed discussion of the results is provided in the following discussion section.

### Results of transfer learning networks

The results of experiments using different hyperparameters values are shown and the best settings for each one are selected. The format of the model column of the provided tables is as follows: Model Name, Learning Rate, With decay or No decay, Dropout Percentage, and Batch Size.

The first network, “VGG16”, is discussed and shown in Table 5. From our experiments, it is noticed that decreasing the learning rate while applying the decay rate yields better results. Furthermore, choosing a higher dropout percentage reduces the differences between the validation loss and testing loss which reduces the effects of overfitting. Finally, increasing or decreasing the batch size caused the network to misclassify some of the images. In conclusion, the best set of hyperparameters for the VGG16 network is found to be using 0.0001 as the learning rate with applying decay on it, 0.5 dropout percentage, and a batch size of 64.

**Table 5:** VGG resutls.

Model	Training accuracy (%)	Training loss	Validation accuracy (%)	Validation loss	Testing accuracy (%)	Testing loss
VGG16_0.001_WD_0.5_64	98.93	0.03	98	0.05	97.28	0.09
VGG16_0.001_WD_0.2_64	98.28	0.05	98.75	0.05	96.88	0.1
VGG16_0.001_ND_0.5_64	97.27	0.09	97.25	0.09	96.65	0.1
VGG16_0.001_WD_0.5_64	98.66	0.04	98.00	0.04	97.60	0.09
VGG16_0.0001_ND_0.2_64	99.05	0.04	97.75	0.08	97.28	0.09
VGG16_0.0001_WD_0.2_64	99.16	0.04	97.50	0.07	97.04	0.11
VGG16_0.0001_ND_0.5_64	99.75	0.01	97.50	0.08	97.52	0.09
VGG16_0.0001_WD_0.5_64	99.06	0.03	97.25	0.06	98.00	0.07
VGG16_0.0001_WD_0.5_32	98.21	0.06	97.75	0.09	97.20	0.09

The next networks that are taken into consideration are ResNet50 shown in Table 6, and InceptionV3 presented in Table 7. These two networks are discussed together as they have the same behavior and best set of hyperparameters. The same behavior of adjusting the learning rate and decay is observed. On the other hand, in ResNet50 choosing a lower dropout percentage and batch size improved the performance of the network significantly. Thus, the best set of hyperparameters for the ResNet50 and InceptionV3 networks is found to be using 0.0001 as the learning rate with applying decay on it, 0.2 dropout percentage, and batch size of 32.

**Table 6:** ResNet50 results.

Model	Training accuracy (%)	Training loss	Validation accuracy (%)	Validation loss	Testing accuracy (%)	Testing loss
ResNet_0.001_ND_0.2_64	99.62	0.01	99	0.04	98.16	0.11
ResNet_0.001_WD_0.2_64	99.16	0.02	99.25	0.03	98.40	0.09
ResNet_0.001_ND_0.5_64	99.42	0.02	98.75	0.03	98.80	0.08
ResNet_0.001_WD_0.5_64	99.99	0	99.50	0.01	98.88	0.06
ResNet_0.0001_ND_0.2_64	99.76	0.01	100	0.01	98.88	0.03
ResNet_0.0001_WD_0.2_64	99.80	0.01	99.50	0.02	99.12	0.03
ResNet_0.0001_ND_0.5_64	99.56	0.01	99.00	0.03	99.12	0.03
ResNet_0.0001_WD_0.5_64	99.95	0	99.75	0.01	98.96	0.04
ResNet_0.0001_WD_0.2_32	99.99	0	99.50	0.02	99.44	0.02

**Table 7:** InceptionV3 results.

Model	Training accuracy (%)	Training loss	Validation accuracy (%)	Validation loss	Testing accuracy (%)	Testing loss
InceptionV3_0.001_ND_0.2_64	99.34	0.02	99	0.05	98.56	0.04
InceptionV3_0.001_WD_0.2_64	99.12	0.02	99.25	0.03	98.16	0.07
InceptionV3_0.001_ND_0.5_64	98.81	0.04	97.50	0.08	94.33	0.2
InceptionV3_0.001_WD_0.5_64	99.59	0.01	98	0.05	98.40	0.05
InceptionV3_0.0001_ND_0.2_64	99.96	0	99.25	0.03	98.16	0.06
InceptionV3_0.0001_WD_0.2_64	99.86	0	99	0.03	98.24	0.06
InceptionV3_0.0001_ND_0.5_64	99.86	0.01	98.50	0.03	98	0.06
InceptionV3_0.0001_WD_0.5_64	99.89	0.01	99.25	0.03	98.56	0.05
InceptionV3_0.0001_WD_0.5_32	99.34	0.02	99.50	0.02	98.96	0.04

The final network to be examined is DenseNet121 displayed in Table 8. Two sets of hyperparameters are found to give the same results (DenseNet121-0.001-ND-0.5-64 and DenseNet121-0.0001-WD-0.2-64), this observation is proof of the need to study and experiment with different values as modifying some of them results in networks to act in the same manner. That being said, only one set is chosen to be the best set. Even though the two sets yield the same results, when looking at the learning curves of the networks it can be noticed that DenseNet121-0.0001-WD-0.2-64 had more stable learning whereas DenseNet121-0.001-ND-0.5-64 had a lot of ups and drops in the loss during learning.

**Table 8:** DenseNet121 results.

Model	Training accuracy (%)	Training loss	Validation accuracy (%)	Validation loss	Testing accuracy (%)	Testing loss
DenseNet_0.001_ND_0.2_64	99.92	0	99.50	0.01	99.12	0.03
DenseNet_0.001_WD_0.2_64	99.54	0.01	99.50	0.04	98.24	0.05
DenseNet_0.001_ND_0.5_64	99.99	0	99.50	0.02	99.44	0.02
DenseNet_0.001_WD_0.5_64	99.22	0.02	99.25	0.04	98.80	0.05
DenseNet_0.001_ND_0.5_32	99.24	0.02	99.50	0.01	98.56	0.09
DenseNet_0.001_ND_0.5_128	99.69	0.01	99.50	0.02	98.72	0.1
DenseNet_0.0001_ND_0.2_64	99.94	0	99.75	0	99.28	0.03
DenseNet_0.0001_WD_0.2_64	99.93	0	99.50	0.01	99.36	0.02

Thus, we conclude that the best set of hyperparameters for the DenseNet121 network is found to be using 0.0001 as the learning rate with applying decay on it, 0.2 dropout percentage, and a batch size of 64 is reached. Further explanations are found in Table 9, which represents the best set of values for hyperparameters of each network is provided.

**Table 9:** Best set of hyper parameters.

Model	Learning rate	Decay (W/N)	Dropout	Batch size
VGG16	0.0001	W	0.5	64
ResNet50	0.0001	W	0.5	32
InceptionV3	0.0001	W	0.5	32
DenseNet121	0.0001	W	0.2	64

### Comparisons of different networks

As shown above, the best-performing networks are compared to nominate the best network based on our dataset and experiments to be used in other applications. Table 10 summarizes the results of all the experiments and presents the best-performing network of each type.

**Table 10:** Comparison of best performance models.

Model	Training accuracy (%)	Training loss	Validation accuracy (%)	Validation loss	Testing accuracy (%)	Testing loss
VGG16_0.0001_WD_0.5_64	99.06	0.03	97.25	0.06	98.00	0.07
ResNet_0.0001_WD_0.2_32	99.99	0	99.50	0.02	99.44	0.02
InceptionV3_0.0001_WD_0.5_32	99.34	0.02	99.50	0.02	98.96	0.04
DenseNet_0.0001_WD_0.2_64	99.93	0	99.50	0.01	99.36	0.02

**Figure 4:**
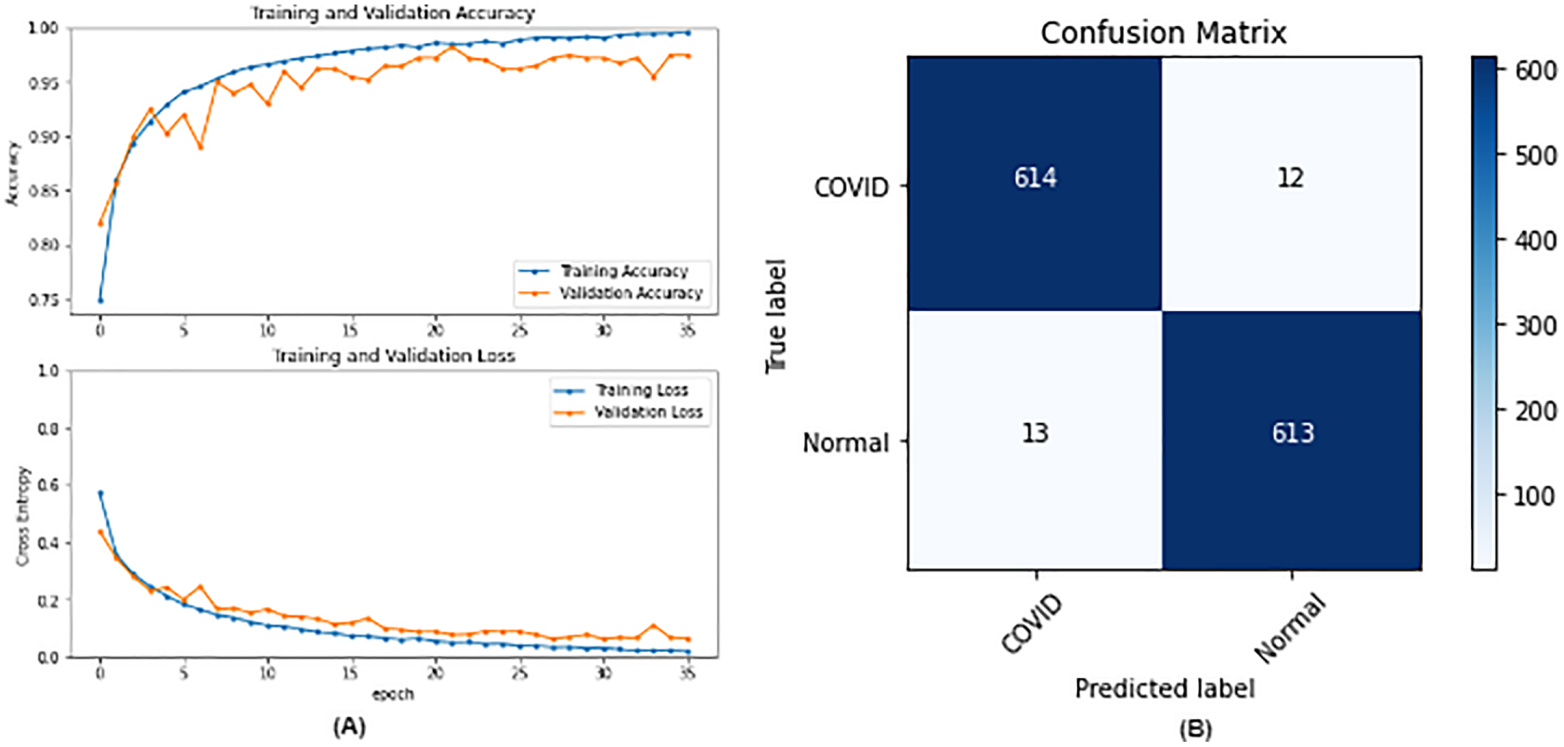
VGG16 results where sub-figure (A) represents the learning curve of the model, while sub-figure (B) illustrates the confusion matrix on the testing data.

**Figure 5:**
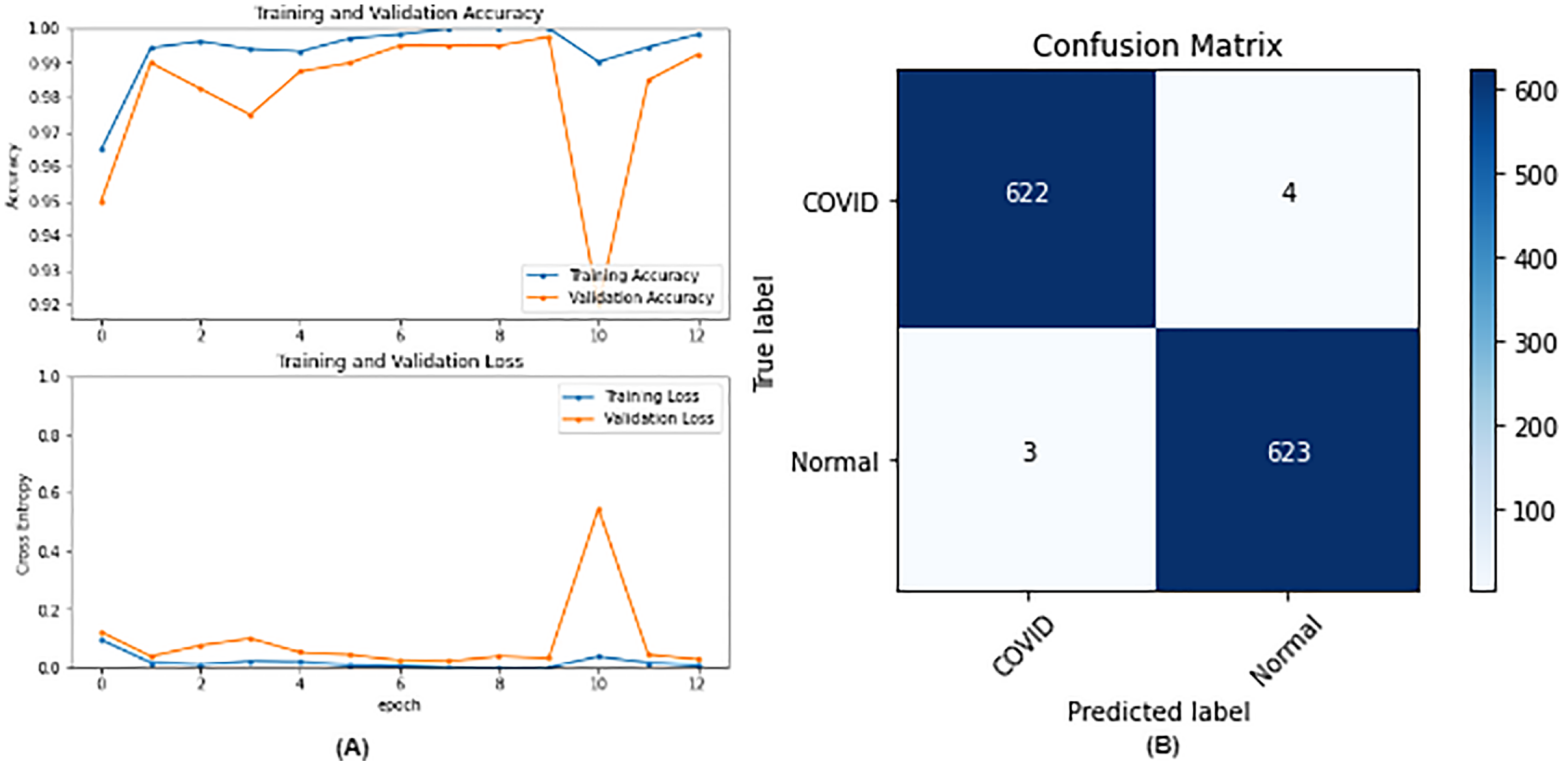
ResNet50 results where sub-figure (A) represents the learning curve of the model, while sub-figure (B) illustrates the confusion matrix on the testing data.

**Figure 6:**
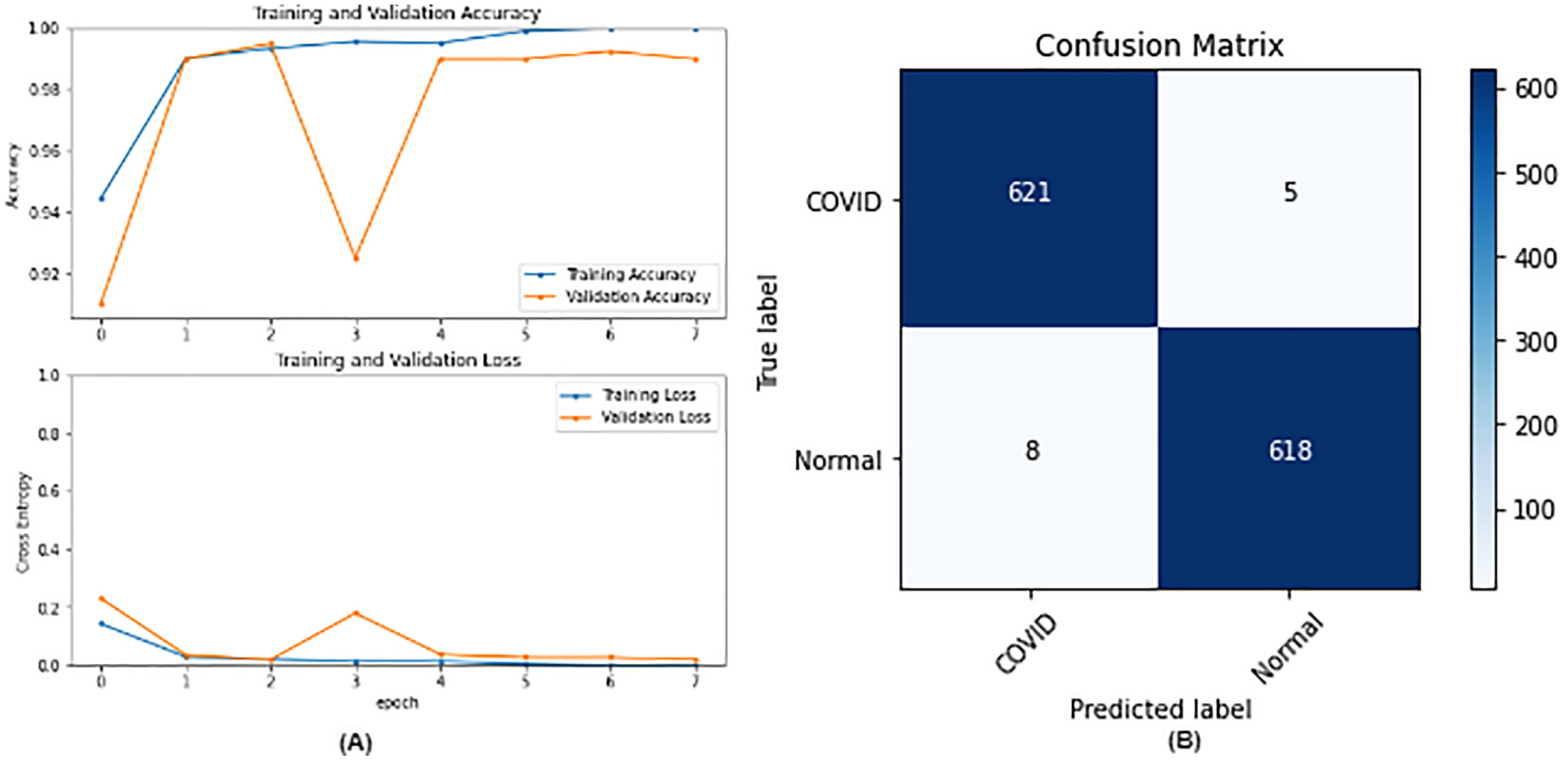
IncetionV3 results where sub-figure (A) represents the learning curve of the model, while sub-figure (B) illustrates the confusion matrix on the testing data.

**Figure 7:**
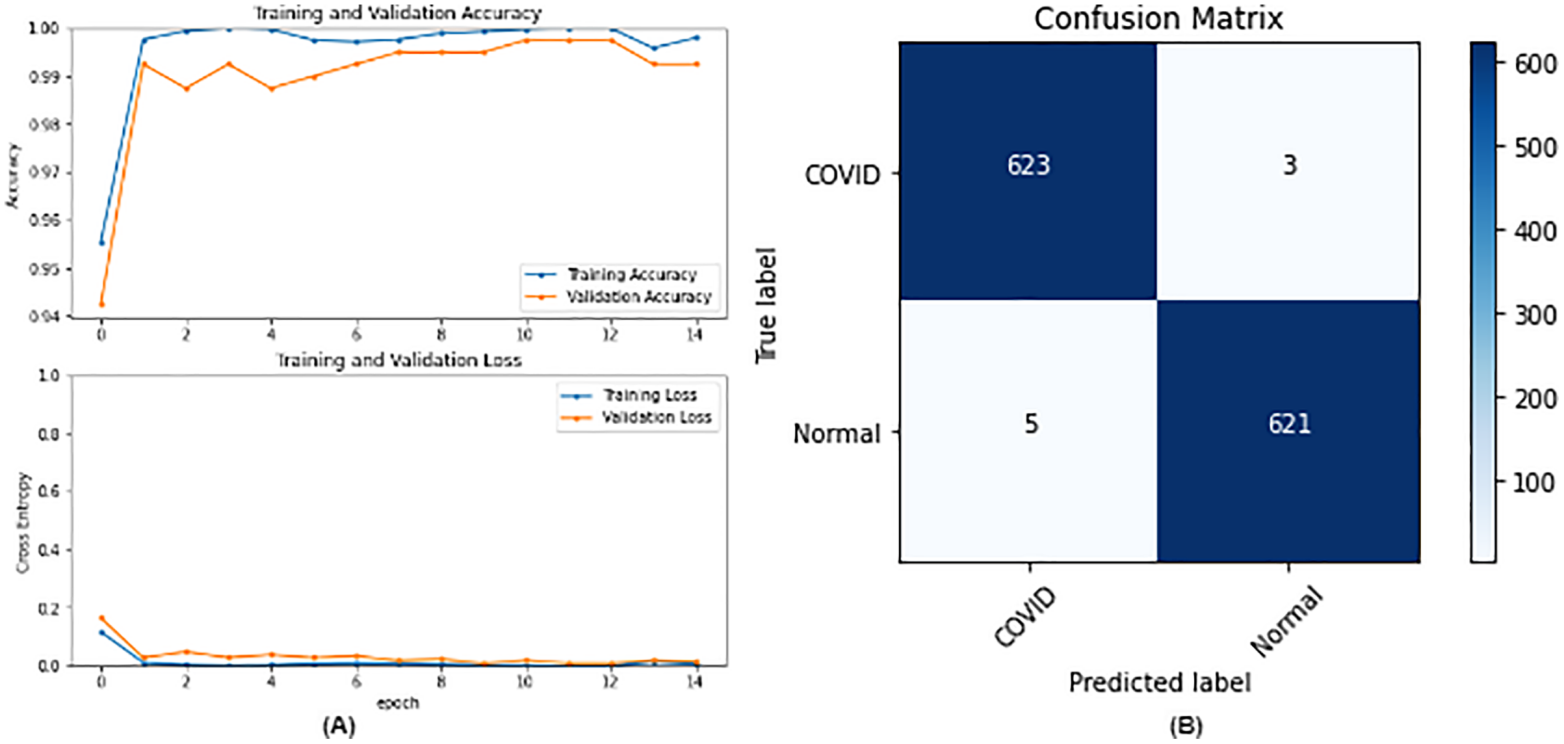
DenseNet121 results where sub-figure (A) represents the learning curve of the model, while sub-figure (B) illustrates the confusion matrix on the testing data.

From the table, it is noticed that ResNet50 and DenseNet121 have very close results and outperform VGG16 and InceptionV3. The two networks have impressive results, and both can be used in other applications. But in our work, only one is chosen to be the best-fitted network. If confusion matrix data is considered, ResNet50 misclassifies seven images whereas DenseNet121 misclassifies eight images. Thus, ResNet50 is nominated to be the most suitable network after conducting the discussed experiments and comparisons of the figures that depict the (a) learning curves and (b) confusion matrix for each one of the best-performing models. For example, Fig. 4 represents VGG16 results, whereas Figs. 5–7 represent the results for ResNet50, InceptionV3, and DenseNet121 respectively.

In our case, the false positives are represented by the top right corner of the confusion matrix figure where they refer to patients that do not have COVID but are positively classified. This could lead to a problem of shortage of medical supplies if they are hospitalized. As for the false negatives that are represented in the bottom left corner, they refer to patients with COVID that are classified as non-COVID. This could lead to a huge problem if they are not quarantined and are allowed to walk freely with crowds. Thus, the best-performing model is the one with the minimum number of FP and FN. In order to verify the validity of our findings, the results of our work is compared with several previous works. As shown in Table 11, the results of our models outperform all previously developed models included in the literature of this article.

**Table 11:** Comparison with previous work.

Paper	Dataset	Models	Best result (Accuracy)
Chakraborty, Murali & Mitra (2022)	COVID-19 and Pneumonia x-rays	ResNet18	DenseNet (96.43%)
		AlexNet	
		DenseNet	
		VGG16	
Yadlapalli et al. (2022)	COVID19-CT	VGG16	VGG16 (89%)
		ResNet50	
		InceptionV3	
Padma & Kumari (2020)	COVID chest Xray	2D CNN	Training ACC: 99%
			Validation ACC: 98.3%
Khan et al. (2022)	BIMCV- COVID19+	EfficientNetB1	EfficientNetB1 (96.3%)
		NasNetMobile	
		MobileNetV2	
Aggarwal et al. (2022)	Two datasets	MobileNetV2	
		Xception	
		ResNet50V2	Dataset 1:
		DenseNet121	DenseNet121 (97%)
		inceptionResNetV2	Dataset 2:
		VGG19	MobileNetV2 (85%)
		NASNetMobile	
		Inceptionv3	
Nazish et al. (2021)	COVID-19 patients x-rays	Logistic Regression	SVM (96%)
		SVM	
Present study	COVID-19 radiography database	VGG16	ResNet50
		ResNet50	Training ACC: 99.99%
		InceptionV3	Validation ACC: 99.50%
		DenseNet121	Testing ACC: 99.44%

## Discussions and limitations

Further explanations and discussions are presented. Prior research has shown that DenseNet121 is a good choice for x-ray images as noticed in our work. Furthermore, they stated that InceptionV3 is a good choice but neglected the performance of ResNet50 even though it is used in their work. This might be due to not experimenting with enough hyperparameters to reveal the quality of its performance which has been shown in our work. VGG16 got the lowest results which are normal because of its shallow architecture when compared to the other architectures.

On the other hand, InceptionV3 has 48 layers close to the number of layers of ResNet50 which is 50. Thus, our experiments have shown that the residual block technique is more useful than the inception module technique which is introduced by InceptionV3.

Finally, DenseNet121 which has 121 layers has achieved close to ResNet50 which has far fewer layers. This could be due to the overparameterization problem which means that increasing the number of layers does not necessarily improve the results. Furthermore, training 50 layers take far less time compared with training all 121 layers of the DenseNet.

**Figure 8:**
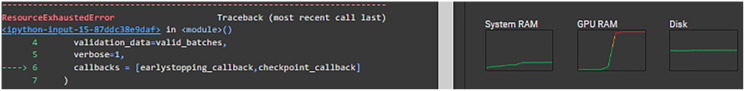
Resource exhausted error.

For the limitations, Google Collaboratory was used in this research which offers free usage of GPU, which was needed in this research to enhance the speed of execution, but with limited time per day and a limited number of sessions in the free model. Furthermore, some configurations could not be executed due to “ResourceExhaustedError” in Fig. 8. The runtime was long even with the GPU and because our research included running a lot of experiments it as difficult to do. Even though data augmentation has shown improvements with image classification but in our case, it adversely affected the results. This could be attributed to the fact that general image augmentation techniques may not be suitable for medical images. This is an interesting research question that is worth investigating in the future.

## Conclusion and future work

The purpose of this research was to train different networks on the lung X-rays dataset to classify whether the patient has COVID-19 or not, and conduct comparisons between them to help the medical field during the faced pandemic. Future work includes exploring segmentation (Zaitoun & Aqel, 2015) techniques to train the model only on the interesting and important parts of the images, which could improve the results. Furthermore, using hybrid approaches through implementing recurrent neural networks (RNN) (Sherstinsky, 2020) and transformers along with attention techniques (Vaswani et al., 2017) should be taken into consideration as it is considered a rising topic these days. Different approaches used by the researchers are noticed, but mainly most of them based their work on transfer learning. This is understandable as building a network from scratch requires a lot of data and is time-consuming. Nevertheless, with enough data, building a model from scratch is applicable and might lead to better results as it is built and trained for a specific task. Future work may include building a network from scratch and conducting a comparison between the results of transfer learning networks and the network that is built from scratch. Finally, further experiments could also be done to find the best parameters for data augmentation and assess its role in medical images.

## Supplemental Information

10.7717/peerj-cs.1082/supp-1 Supplemental Information 1 COVID Sampling Code.

10.7717/peerj-cs.1082/supp-2 Supplemental Information 2 pre-processing code.

10.7717/peerj-cs.1082/supp-3 Supplemental Information 3 DenseNet Code.

10.7717/peerj-cs.1082/supp-4 Supplemental Information 4 ResNet Code.

10.7717/peerj-cs.1082/supp-5 Supplemental Information 5 VGG Code.

10.7717/peerj-cs.1082/supp-6 Supplemental Information 6 Inception Code.

## Additional Information and Declarations

### Data Availability

The following information was supplied regarding data availability: The two raw datasets are available on Kaggle: – COVID-19 Radiography Dataset, https://www.kaggle.com/datasets/preetviradiya/covid19-radiography-dataset. – Covid-19 X-ray – Two proposed Databases, https://www.kaggle.com/datasets/edoardovantaggiato/covid19-xray-two-proposed-databases.
